# Gut microbiota is associated with spatial memory and seed-hoarding behavior of South China field mice (*Apodemus draco*)

**DOI:** 10.3389/fmicb.2023.1236359

**Published:** 2023-09-13

**Authors:** Enping Feng, Xifu Yang, Kunming Zhao, Ying Li, Hanyi Zhu, Zhenshan Wang, Zhibin Zhang

**Affiliations:** ^1^College of Life Science, Hebei University, Baoding, Hebei Province, China; ^2^State Key Laboratory of Integrated Management on Pest Insects and Rodents, Institute of Zoology, Chinese Academy of Sciences, Beijing, China; ^3^College of Ecology and Environment, Chengdu University of Technology, Chengdu, China; ^4^College of Life Science, University of Chinese Academy of Sciences, Beijing, China; ^5^CAS Center for Excellence in Biotic Interactions, University of Chinese Academy of Sciences, Beijing, China

**Keywords:** seed hoarding, spatial memory, gut microbiota, fecal microbiota transplantation (FMT), rodents

## Abstract

**Background:**

Scatter-hoarding animals store food in multiple locations within their home range and rely on spatial memory for subsequent localization and retrieval. The relationship between memory and scatter-hoarding behavior has been widely demonstrated, but the association of gut microbiota with spatial memory and seed-hoarding behavior of animals remains unclear.

**Methods:**

In this study, by using enclosure behavior tests, memory tests including an object location test (OLT) and a novel object recognition test (NORT), and fecal microbiota transplantation (FMT) experiment, we evaluated the role of gut microbiota in affecting the memory and seed-hoarding behavior of rodents. According to their scatter-hoarding intensity, South China field mice (*Apodemus draco*) were divided into scatter-hoarding group (SG) and non-scatter-hoarding group (NG).

**Results:**

We found that the SG performed better than the NG in the NORT. FMT from SG donor mice altered the NG recipient mice’s gut microbiota structure. Further tests demonstrated FMT from SG donor mice increased memory of NG recipient mice in laboratory tests and seed larder hoarding intensity of NG recipient mice in enclosures.

**Conclusion:**

Our results suggest gut microbiota could modulate the memory and seed-hoarding behavior of animals.

## Introduction

Animals living in seasonally changing environments are faced with uncertain variety in food abundance and availability (Vander Wall, 1990; Liu et al., 2022; Yu et al., 2022). As a result, some species evolved a crucial adaptive strategy of food hoarding to cope with the dynamic in the environment (Vander Wall, 1990; Zeng et al., 2021). In general, hoarding behaviors can be divided into two categories: scatter-hoarding (food items are dispersed and hoarded in many different locations) and larder-hoarding (all food is stored in one or a few locations or nests) (Preston and Jacobs, 2001; Brodin, 2010). Employing the larder-hoarding strategy would counter the risk of losing all food supplies, which must be defended to prevent competitors or pilfers. Instead, scatter-hoarded food is protected by hiding them in multiple locations with a low storage density and these food caches do not need much defense, but need better spatial memory for late relocation. Many rodent species have been reported with scatter-hoarding behavior, such as Siberian chipmunk (*Tamias sibiricus*), Pallas’s Squirrel (*Callosciurus erythraeus*), Edward’s long-tailed rats (*Leopoldamys edwardsi*), and South China field mice (*Apodemus draco*) (Pan et al., 2013; Yi S et al., 2021; An et al., 2022; Xiao et al., 2022).

As a result of scatter-hoarding, numerous food caches are created by animals during autumn (fruit ripening) to provide a sufficient and predictable food supply during winter (Cao et al., 2016; Yang et al., 2020). Making a large number of caches represents a heavy investment in time and resources during scatter hoarding. Therefore, the high reliance on scatter-hoarding might suggest that spatial memory should be enforced in scatter-hoarding species as a result of selection pressure on memory (Pan et al., 2013; Yi X et al., 2021). For example, Pan et al. (2013) have demonstrated that hippocampal cells, which are strongly associated with spatial memory, proliferated more in scatter-hoarding rodents than in non-hoarding ones. The long-term retention of cache memory was found to vary separately from weeks to months depending on species (Pravosudov and Roth, 2013). Although the relationship between spatial memory and scatter-hoarding behavior has been demonstrated in many studies (Vander Wall, 1982, 1991; Smith and Reichman, 1984; Shettleworth, 2010; Wang et al., 2018; Yi X et al., 2021), the underlying mechanism was rarely investigated. In addition, previous studies mostly focused on the role of the brain, especially the hippocampus, the brain region essential for spatial memory (Krebs et al., 1989; Pan et al., 2013; Pravosudov and Roth, 2013).

Gut microbiota consists of bacteria, fungi, protozoa, and archaea in the gastrointestinal tract and their interactions with their hosts have been extensively studied (Shreiner et al., 2015). Gut microbiota is involved in a variety of behavioral and physiological processes of the host, including the immune system, cardiovascular and cerebrovascular disease, obesity, and brain function (Sampson and Mazmanian, 2015; Fung et al., 2017; Ascher and Reinhardt, 2018; Virtue et al., 2019). Moreover, increasing evidence shows that gut microbiota plays an essential role in brain memory (Mayer, 2011). The gut-brain axis, a bilateral communication system between the gut and brain, could provide gut microbiota and its metabolites access to the brain through neural, hormonal, and immunological signals (Collins et al., 2012). It was reported that long-term *Lactobacillus* and *Bifidobacterium* dietary supplements can enhance rats’ performances in watermaze spatial navigation and long-term object recognition memory (O’Hagan et al., 2017). *Citrobacter rodentium* infection in mice was found to cause stress-induced memory dysfunction (in particular, concerning discrimination of novel objects and T-maze performance), which could be resolved by treatment with probiotics (Gareau et al., 2011). Furthermore, Wang et al. (2018) have found that improved memory can facilitate scatter-hoarding ability. Consequently, we hypothesized that gut microbiota may be associated with spatial memory and seed-hoarding behavior of rodents via the gut-brain axis.

South China field mouse (*Apodemus draco*) is a seed-eater rodent species inhabiting forests in southern China (Yang et al., 2022a, 2023). *A. draco* exhibits both seed scatter-hoarding and larder-hoarding behaviors (Chang et al., 2009). In this study, we examined the association of gut microbiota with spatial memory and seed-hoarding behavior of this species, aiming to test the above hypothesis. We have the following predictions: (1) Mice which scatter hoarded more seeds should own a better spatial memory and (2) Fecal microbiota transplantation from donor mice with better scatter-hoarding ability could enhance recipients’ spatial memory and scatter-hoarding intensity.

## Materials and methods

### Study site

This study was conducted in a subtropical evergreen broad-leaved forest of Dujiangyan City (altitude 600–1,000 m, 31°04′N-31°05′N, 103°42′E-103°42′E), Sichuan Province, China. The local annual mean temperature is 15.2°C, and annual precipitation is 1,200–1,800 mm. The climate is often cloudy with few sunny days. The major rodent species include *Apodemus draco*, *Apodemus chevrieri*, *Niviventer fulvescens*, *Niviventer niviventer*, and *Leopoldamys edwardsi* (Yang et al., 2018, 2022b).

### Study animals

Wire live traps (L × W × H = 30 cm × 13 cm × 12 cm), baited with fresh chestnuts, were used to capture small rodents. Following Yang et al. (2018), we set 4 × 10 trapping grids with an interval of 10 m in each plot. Traps were settled at 14:00–16:00 in the afternoon and checked at 08:00–10:00 the next morning. Captured rodents were identified to species and age. Then we recorded sex, body mass, and reproductive status (females pregnant, lactating or not; males with testes descended or not).

Forty-eight males and twenty-eight females of captured *A. draco* were selected. Prior to the experiments, *A. draco* was housed in plastic cages individually (L × W × H = 30 cm × 15 cm × 20 cm) with sawdust bedding and provided with rat chow (Shenyang Maohua Biotechnology Co. Ltd., Liaoning, China) and tap water *ad libitum*. The temperature of the feeding chamber was maintained at 20–25°C. Cotton was added for insulation in winter. All subjects were exposed to a 14 h light / 10 h dark photoperiod. About 4 months passed from the day of the capture of mice until the end of the tests. All animals were housed in cages under laboratory conditions and fed with rat chow once captured from the field. The numbers of animals used in each experiment and category were shown in Supplementary Table S1.

### Experimental enclosures

In this study, there were five larger experimental enclosures (L × W × H = 10 m × 10 m × 2.5 m; see Yi X et al., 2021 for details). The top of the enclosures was covered by iron sheets to prevent any predators from entering the enclosure. We divided each of the five larger enclosures by using 0.6 m high iron sheets to obtain four enclosures (Supplementary Figure S1). Thus, a total of 20 enclosures (L × W × H = 5 m × 5 m × 0.6 m) were used to evaluate the scatter-hoarding intensity of the rodents. We reinforced the separation walls with bricks settled on the wall bottom and iron wires linked to the ceiling. In each enclosure, the floor was built of bricks, and contained 16 shallow pits (L × W × D = 24 cm × 12 m × 6 cm) separated by 1 m to simplify the experimental design by referring to Yi X et al. (2021). All pits were filled with sand for rodents to cache seeds. One nesting box (L × W × H = 40 cm × 40 cm × 40 cm) and a plate were placed at the same corner of each enclosure to allow rodents to rest and drink freely. A central area of 0.5 m × 0.5 m of each enclosure was settled as the seed station.

### Scatter-hoarding intensity test

All subjects were transported individually to the enclosure for the scatter-hoarding intensity test. An obligatory acclimation for one night was conducted for all animals before the tests. Animals were provided with chestnuts and water *ad libitum*. After acclimatization, we cleared away the uneaten food at 08:00 to ensure that the animals were starved for 8 h. Chestnuts were used in this study and tagged with a numbered plastic tag for relocation and identification. Before seed release, we drilled a 0.3-mm hole through the husk of chestnuts and tied the numbered plastic tag with a thin 10 cm long steel thread through the hole. At 16:00, 20 labeled chestnuts were placed in the seed station. Subjects were allowed to interact freely with the seeds until the next morning. At 08:00 the next morning, subjects were moved out of enclosures, and all seed fates were recorded (intact *in situ*, IIS; eaten *in situ*, EIS; scatter hoarded, SH; larder hoarded, LH; eaten after removal, EAR; eaten in the nest, EIN; intact after removal, IAR) (Wang and Yi, 2022). After each experiment, the enclosures were cleaned and ventilated for at least one night. The enclosure test was performed three times for each subject to achieve reliable data.

By referring to a previous study (Pan et al., 2013), the subjects were divided into scatter-hoarding group (SG) or non-scatter-hoarding group (NG) according to the result of the scatter-hoarding intensity test. The SG is comprised of the rodents that scatter-hoarded ≥5% of seeds at least once in three-time tests, while the NG is composed of rodents that scatter-hoarded <5% of the seeds. The scatter-hoarding intensity was evaluated according to the number of SH seeds. Because scatter hoarding behavior is often disrupted in enclosure tests, thus, we also referred to the other behaviors in defining the individuals of SG and NG groups. In this work, we observed that individuals adopting scatter hoarding strategy also had a larger hoarding intensity, but less eating intensity *in situ* or after seed removal as compared to the NG group.

### Memory test

Following Denninger et al. (2018), we set four testing arenas (L × W × H = 40 cm × 40 m × 40 cm) in a 2-by-2 manner on the floor with environmental cues arranged on trilateral sides (Supplementary Figure S2). A camera was settled above the arenas, about 1.7 m high to the floor. All procedures were recorded on video.

To evaluate the memory, we performed a memory test consisting of an object location test (OLT) and a novel object recognition test (NORT). Animal habituation, training, and testing of the OLT and the NORT were performed under the instruction of Denninger et al. (2018). All animals under test were numbered and the test order was arranged randomly. The inter-trial intervals (ITI) were extended to 24 h to assess the long-term memory (contrary to short-term memory which lasts for about 20 s, the retention of long-term memory lasts from minutes to multiple years) of rodents. The 4-day experiment was conducted from 08:00 to 18:00. We brought animals to the test room for a 30-min acclimation before starting the test.

**Figure 1:**
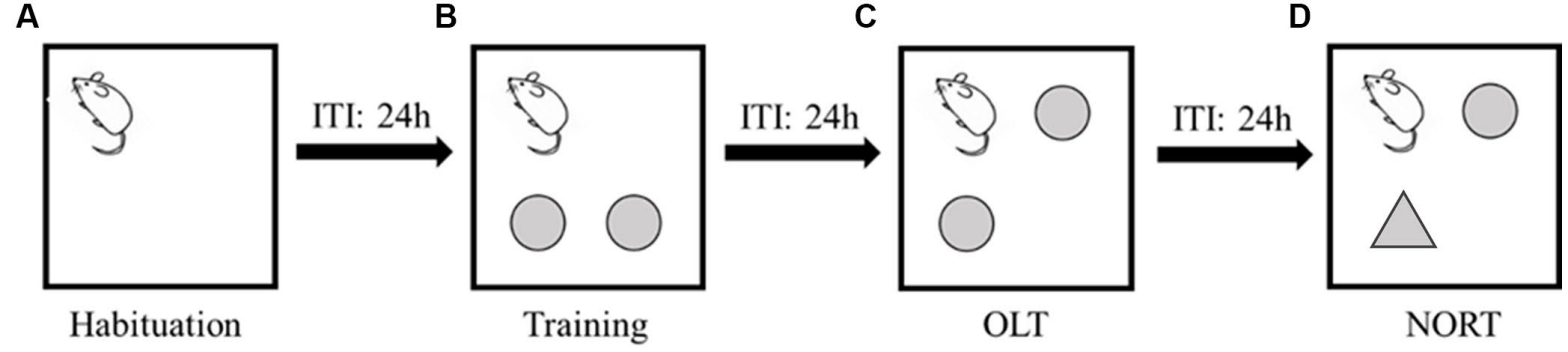
Procedures of memory test with inter-trial intervals (ITI) of 24 h. **(A)** Habituation session. The square represents the arena and the corner mouse faced is the release corner. **(B)** Training trial. The two circles represent the objects. **(C)** Object location test (OLT). The upper right circle represents one object that was moved to a novel location. **(D)** Novel object recognition test (NORT). The triangle represents the novel object.

During the habituation session, mice were placed in the arenas (one mouse per arena) faced to the release corner (Figure 1A) and allowed to explore the arenas freely for 10 min. In the training trial, two objects were affixed 12 cm × 12 cm away from 2 non-release corners, and mice were placed and faced the same release corner (Figure 1B) and were able to explore the environment for 10 min. In the OLT, one of the two objects in the training trial was moved to another non-release corner (Figure 1C), and mice were allowed for a 10-min exploration. In the NORT, we used a novel object to replace the object that was not moved in the OLT (Figure 1D) and repeated the 10-min exploration test.

After each test, mice were sent back to their housing cages individually and all arenas were cleaned with 75% ethanol to minimize olfactory cues. All procedures were video recorded by an overhead camera. The movements were tracked and assessed with EthoVision® XT (Noldus, Wageningen, Netherlands). The valid investigation time was scored when the subject pointed its nose at the object at a maximum distance of 2 cm from that object. The time that the subject climbed on or jumped off the object was excluded. Moreover, the mice that did not investigate the objects were excluded from the analyses since the memory test was designed by allowing the mice to explore and remember the original objects in previous trials and recognize which object was removed or replaced in the following trials. The percentage of investigation time was calculated as $100\times \left[t_{\mathrm{novel}}/\left(t_{\mathrm{novel}}+t_{\mathrm{similar}}\right)\right]$ . Values above 50 indicate preferences for object location or novel object.

### Fecal microbiota transplantation

To investigate whether the gut microbiota could mediate memory and the intensity of scatter-hoarding of rodents, FMT was conducted to alter the structure and function of the gut microbiota of the recipient animals. The SG and NG groups were selected as the donors and recipients, respectively. Fresh feces from donors (100 mg) were resuspended in 1 mL of sterile 0.9% saline by vortexing for 5 min. Then we centrifuged the supernatant (500 g, 1 min) to prepare a bacterial suspension. Forty-three NG mice were used as recipients (22 males and 21 females), which were randomly divided into two groups: the FMT treatment group NG-SG (11 males and 10 females) received bacterial suspension from SG donors, while the control group NG-NS (11 males and 11 females) received sterile 0.9% saline. Animals of both recipient groups were first treated with 200 μL antibiotics (100 μg/mL neomycin, 50 μg/mL streptomycin, and 100 U/mL penicillin) via intragastric gavage for successive 3 days of administration. After antibiotic treatment, the NG-SG and NG-NS groups were administered by gavage with 200 μL bacterial suspension from SG donors and sterile saline for 30 days every 3 days, respectively. After FMT, the scatter-hoarding intensity test and memory test were conducted to evaluate the differences in hoarding behavior and memory among the NG-SG and NG-NS groups. For fecal collection, mice were placed in sterilized cages (L × W × H = 30 cm × 15 cm × 20 cm). At the same time, fresh feces were collected from each mouse within 10 min with forceps and then stored at −80°C for subsequent 16S rRNA analysis (Zhu et al., 2022). After sample collection, the mice were returned to their cages.

### 16S rRNA profiling

Total genome DNA from samples was extracted using the cetyltrimethylammonium bromide (CTAB) method. The purity and concentration of DNA were evaluated on 1% agarose gels. Then DNA was diluted to 1 ng/μL with sterile water. Forward primer 341F (5’-CCTAYGGGRBGCASCAG-3′) and reverse primer 806R (5’-GGACTACNNGGGTATCTAAT-3′) were adopted for amplification of the V3–V4 regions of 16S rRNA gene. The library was generated using NEB Next^®^ Ultra DNA Library Prep Kit (Illumina, United States) and added with index codes. The quality of the library was assessed using Agilent 5,400 (Agilent Technologies Co Ltd., United States). Finally, the library was sequenced on the Illumina NovaSeq platform and 250 bp paired-end reads were generated. Sequencing services were provided by Wekemo Tech Group Co., Ltd. Shenzhen China.

### Bioinformatics processing

Demultiplexed sequences were filtered for quality, then trimmed, de-noised, and merged with the Quantitative Insights Into Microbial Ecology (QIIME, version 2) software suite (Callahan et al., 2016). DADA2 plugin was used to obtain the feature table of the amplicon sequence variants (ASVs) by removing the chimeric sequences. Taxonomic classification of ASVs was conducted in QIIME2 using classify-sklearn with a pre-trained Greengenes (13_8 release) 99% database with a confidence threshold of 0.7 (Bokulich et al., 2018). Diversity metrics were generated from the ASVs feature table using the core-diversity plugin within QIIME2. The minimum sampling depth is 60,217 reads in our study. Microbial community of different relative abundance among samples and groups was identified with appropriate methods, including analysis of the composition of microbiome (ANCOM), and linear discriminant analysis (LDA) Effect Size (LEfSe) while the DESeq2 analysis was based on absolute abundance (Segata et al., 2011; Love et al., 2014; Mandal et al., 2015).

### Statistical analysis

Data were analyzed using R software. Kolmogorov–Smirnov tests were used to assess the variance and normality of data on seed fates and memory tests. Data on seed fates were not normally distributed, therefore we used generalized estimating equations (GEE; R package “gee”; category × sex; Zeger et al., 1988) to analyze the differences in seed fates between groups. As the data on memory were normally distributed, the difference of groups in memory tests was evaluated using two-way ANOVA (category × sex). Alpha diversity was assessed in QIIME2 by the Kruskal-Wallis test applied to the Chao1, Observed, Shannon, and Simpson indices calculated from the obtained ASVs. Rarefaction curves were calculated using the Observed index at the ASV level with OriginPro (Version 2023, OriginLab Corporation, Northampton, MA, United States). Based on the Bray-Curtis distance, permutational multivariate ANOVA (PERMANOVA) was used to evaluate the differences between groups in beta diversity (adonis, permutation = 9,999). The results of beta diversity were visualized via nonmetric multidimensional scaling (NMDS) and partial least squares discriminant analysis (PLS-DA) with the “mixOmics” package in R software (Rohart et al., 2017). The level of statistical significance was set at *p* < 0.05. We focused on analyses of differences between SG and NG, and between NG-SG and NG-NS groups considering the treatment they experienced.

### Ethics statement

Animal raising and handling were in accordance with the guidelines of Animal Use and Care Committee, Institute of Zoology, Chinese Academy of Sciences. Pregnant and immature animals were excluded from the tests, and released immediately on-site. Animals were acclimated to the facility for at least one week before any experiment. All animals were released to the wild after our experiments.

## Results

### Difference in seed fates between the scatter-hoarding group (SG) and non-scatter-hoarding group (NG)

**Figure 2:**
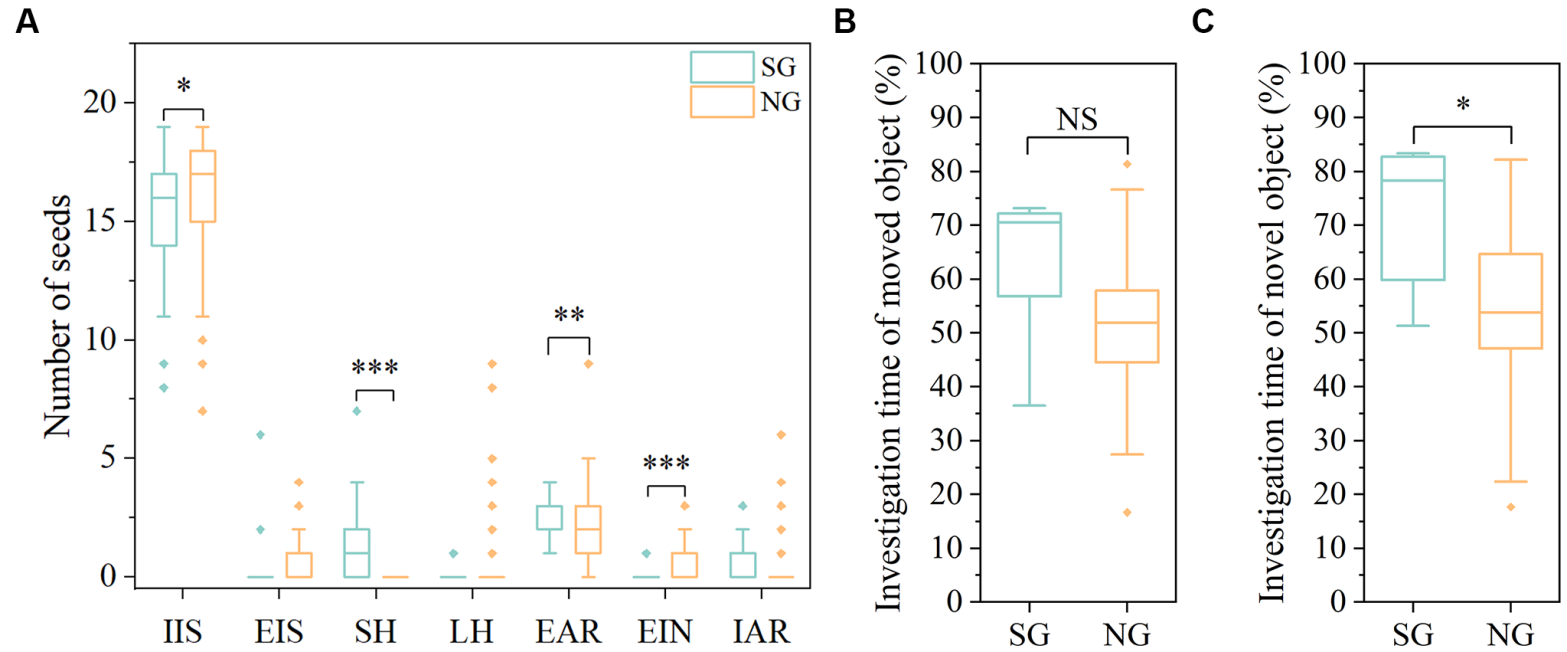
Differences of seed fates and spatial memory between SG and NG. **(A)** Seed fates of SG and NG. **(B)** Percentage of investigation time of the moved object in the object location test (OLT). **(C)** Percentage of investigation time of the novel object in the novel object recognition test (NORT). SG, scatter-hoarding group; NG, non-scatter-hoarding group; IIS, intact *in situ*; EIS, eaten *in situ*; SH, scatter-hoarded; LH, larder-hoarded; EAR, eaten after removal; EIN, eaten in the nest; IAR, intact after removal. Box plots show the median, upper (25%), and lower (25%) quartile of data while dots represent outliers. The medians overlapping with box ranges are not visible in the figures. *p < 0.05, **p < 0.01, ***p < 0.001; NS: *p* > 0.05.

There was a large variation in seed fates between SG and NG. Specifically, compared with the NG, scatter-hoarded intensity (SH; χ^2^ = 14.004, *df* = 1, *p* < 0.001) and the numbers of eaten after removal (EAR; χ^2^ = 7.496, *df* = 1, *p* = 0.006) seeds were significantly increased in SG, while the numbers of intact *in situ* (IIS; χ^2^ = 3.868, *df* = 1, *p* = 0.049) and eaten in the nest (EIN; χ^2^ = 29.122, *df* = 1, *p* < 0.001) seeds were significantly decreased (Figure 2A). There was no significant difference in the numbers of eaten *in situ* (EIS), larder hoarding (LH), and intact after removal (IAR) between SG and NG (all *p* > 0.05). No significant difference between genders and sex-by-group interaction was found in all seed fates (all *p* > 0.05). In total, mice can be divided into two distinct groups in hoarding seeds: SG mice showed higher intensity of scatter hoarding (SH), eaten after removal (EAR), and intact after removal (IAR), but lower intensity of intact *in situ* (IIS), eaten *in situ* (EIS), eaten in the nest (EIN) and larder hoarding (LH) than NG mice.

### Difference in spatial memory between SG and NG

Results of both the object location test (OLT) and novel object recognition test (NORT) showed that the memory of non-scatter-hoarding animals is inferior to that of scatter-hoarding animals (Figures 2B,C). The OLT showed a marginally significant higher preference for the new location in the SG than NG (*F*_(1,51)_ = 3.549, *p* = 0.065; Figure 2B). The NORT results showed that SG spent more time exploring the novel object than the NG (*F*_(1,45)_ = 6.098, *p* = 0.017; Figure 2C). These results indicated scatter-hoarding rodents owned an elevated spatial memory compared to the non-scatter-hoarding rodents. We found there were no significant sex differences and effects of sex-by-group interactions in both OLT and NORT tests (all *p* > 0.05).

### Impacts of FMT on hoarding behavior and spatial memory of rodents

**Figure 3:**
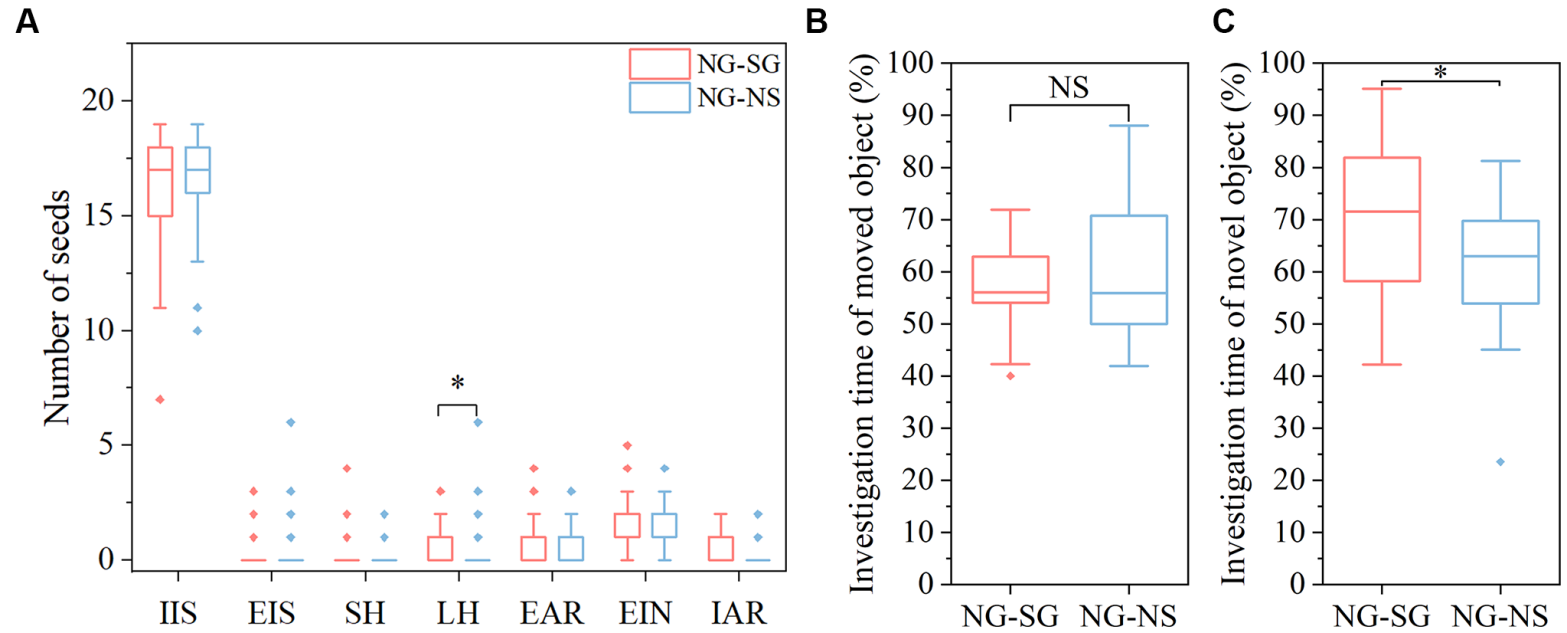
Differences of seed fates and memory between different groups after fecal microbiota transplantation. **(A)** Seed fates in mice of the non-scatter-hoarding group administered with either bacterial suspension from the scatter-hoarding group or sterile 0.9% saline (NG-SG and NG-NS, respectively). **(B)** Percentage of investigation time of the moved object in the object location test. **(C)** Percentage of investigation time of the novel object in the novel object recognition test. IIS, intact *in situ*; EIS, eaten *in situ*; SH, scatter-hoarded; LH, larder-hoarded; EAR, eaten after removal; EIN, eaten in the nest; IAR, intact after removal. Box plots show the median, upper (25%), and lower (25%) quartile of data while dots represent outliers. The medians overlapping with box ranges are not visible in the figures. *p < 0.05; NS: *p* > 0.05.

We found that the NG-SG group (i.e., NG recipients received bacterial suspension from SG donors) larder-hoarded more seeds than the NG-NS group (NG recipients received sterile 0.9% saline) (χ^2^ = 5.418, *df* = 1, *p* = 0.018; Figure 3A). Moreover, the scatter-hoarding intensity was statistically higher in NG-SG group (*p* > 0.05). No significant effects of gender and interaction between sex and group were found in all seed fates (all *p* > 0.05).

A significant FMT effect on memory was observed in the NORT. Compared to the NG-NS group, the members of the NG-SG group showed a significant preference towards the novel object (*F*_(1,31)_ = 4.538, *p* = 0.041; Figure 3C). No FMT effect was found in the OLT (Figure 3B). The results did correspond to the results in the above experiments before FMT and we only found significant variability in the NORT. No sex difference and sex-by-group interaction were found in the OLT and the NORT (all *p* > 0.05).

### Impacts of FMT on the structure and function of gut microbiota

**Figure 4:**
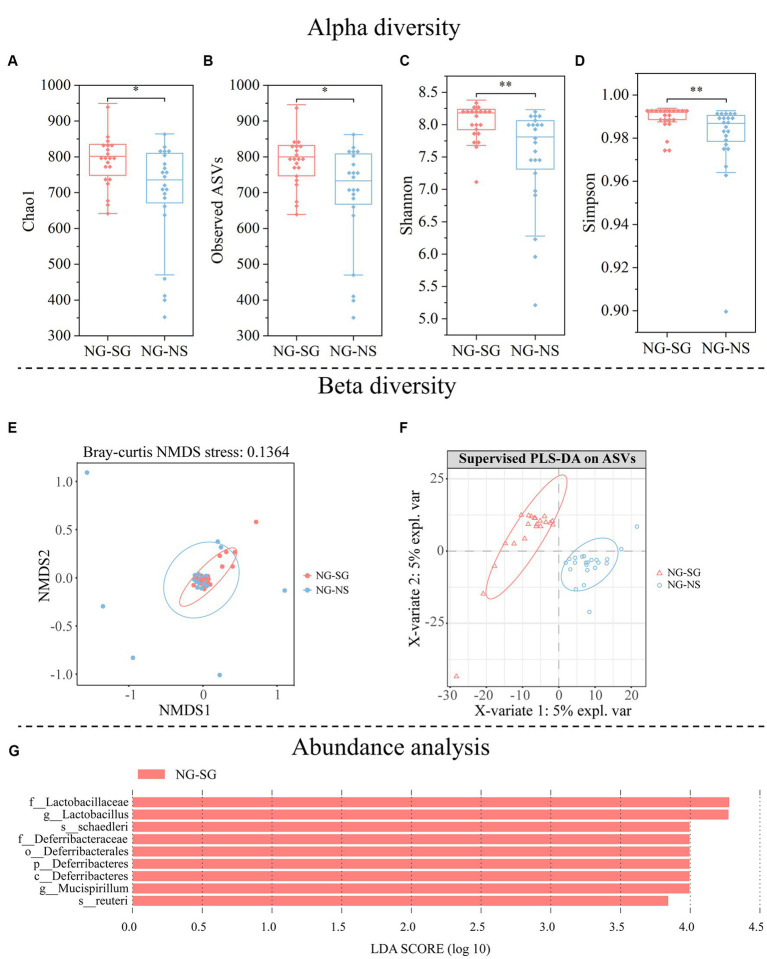
Alpha diversity, beta diversity, and abundance analysis of gut microbiota in mice of the non-scatter-hoarding group administered with either bacterial suspension from the scatter-hoarding group or sterile 0.9% saline (NG-SG and NG-NS, respectively). **(A)** Chao1 diversity. **(B)** Observed-features diversity. **(C)** Shannon diversity. **(D)** Simpson diversity. **(E)** Nonmetric multidimensional scaling (NMDS) plot based on Bray-Crutis distance demonstrating the differences in the microbial community structures of samples from different groups. **(F)** Partial Least Squares Discrimination Analysis (PLS-DA) at the amplicon sequence variants (ASVs) level in different groups. **(G)** The differentially abundant taxa enriched in microbial communities of NG-SG as compared with NG-NS were revealed by linear discriminant analysis (LDA) Effect Size (LEfSe) analysis (LDA score >2). *p < 0.05, **p < 0.01; NS: *p* > 0.05.

The rarefaction curve analysis showed that the microbial diversity across all samples had reached stable (Supplementary Figure S3), indicating that most fecal microbial diversity was captured in our study. Alpha diversity increased in the NG-SG group compared to the NG-NS group (Chao1 index, *p* = 0.027; Observed index, *p* = 0.030; Shannon index, *p* = 0.002; Simpson index, *p* = 0.006; Figures 4A–D). Beta diversity analysis based on Bray-Crutis distance indicated that FMT altered the structure of gut microbiota between the NG-SG group and the NG-NS group (PERMANOVA, *p* = 0.027). The NMDS analysis provided a visual demonstration of the effects of FMT on gut microbiota (Figure 4E), indicating that there were significant differences in the structure of gut microbiota between the NG-SG group and the NG-NS group. Moreover, partial least squares discrimination analysis (PLS-DA) showed that samples were separated with the samples of the NG-SG group occupying the upper left region and the NG-NS group located in the right axis (Figure 4F).

*Firmicutes* was the most abundant bacterial phylum in all groups (NG-SG group, mean = 58.75%; NG-NS group, mean = 54.41%) (Supplementary Figure S4). Compared to the NG-NS group, ANCOM analysis indicated that *Deferribacteres* was enriched at the phylum level (W = 10), and *Deferribacteres* was increased at the class level (W = 9) in the NG-SG group. LEfSE method analysis with LDA score > 2 identified that there were significant differences in the microbial communities among groups (Figure 4G). At the genus level, *Lactobacillus* and *Mucispirillum* were enriched in the NG-SG group. At the species level, *Lactobacillus reuteri* and *Mucispirillum schaedleri* were increased in the NG-SG group. No species were found enriched in the NG-NS group as compared to the NG-SG group.

## Discussion

In this study, we investigated the impacts of gut microbiota in mediating the memory and seed-hoarding behavior of a rodent species (*A. draco*) in a subtropical forest. We divided *A. draco* into two groups according to their seed hoarding behavior such as scatter-hoarding intensity. The scatter-hoarding group (SG) showed a better memory ability than the non-scatter-hoarding group (NG). Fecal microbiota transplantation (FMT) from SG donor mice significantly increased the memory ability of NG-SG recipient mice. The enclosure test results revealed that FMT did not significantly improve the scatter-hoarding ability, but increased the larder-hoarding ability. FMT significantly altered the gut microbiota structure of the NG-SG group. Our results suggest that gut microbiota may play a significant role in mediating the memory ability and seed-hoarding behavior of rodents.

Animals such as scrub jays, rodents, and primates exhibited diverse hoarding behavior (Vander Wall, 1990; Clayton and Dickinson, 1998; Crystal, 2016). It was reported that the cell proliferation in the hippocampus, a brain region essential for memory, increased in the scatter-hoarding rodents compared to the non-scatter-hoarding ones (Pan et al., 2013). Moreover, Zhang et al. (2022) demonstrated an evolutionary relationship between the encephalization quotient (EQ; relative brain size) and hoarding behavior that rodent species with higher EQ are disproportionately likely to scatter hoard. These studies provide both interspecific and intraspecific evidence that rodents could be divided into SG and NG depending on their scatter-hoarding intensity. In this study, our results indicated that the interactions with seeds were more frequent in the SG as the number of intact *in situ* (IIS) seeds, which were left in their original region, was lower in the SG. Along with the elevated numbers of eaten-in-nest (EIN) seeds in NG, it seems that the NG tended to take a more conservative strategy compared to the SG. The potent augment of scatter-hoarded (SH) and eaten after-removal (EAR) seeds in SG provided clues that SG may spend more time away from their home cages which involves a more complex environment. It has been reported that stress could impair hippocampal cell proliferation which is essential for memory while voluntary physical activity and environmental complexity show a positive effect (Lieberwirth et al., 2016). Therefore, the differences in behaviors observed between SG and NG may be linked to variations in animals’ temperament. Bolder animals may be less affected by stress and thus able to plan more intricate routes in which spatial memory plays a crucial role. Based on previous studies (Yi et al., 2016; Yi S et al., 2021), we argued that spatial memory is more essential for the scatter-hoarding behavior of rodents.

Multiple forms of memory are supposed to be involved in food cache and retrieval (Pravosudov and Roth, 2013). A previous study showed that the memory model possesses two basic aspects: source memory and item memory (Slotnick et al., 2000). Source memory refers to memories about conditions in which events were presented, including spatial information (Crystal, 2016). In contrast, item memory focuses on the features of the acquired item or event itself. Thus, we used an object location test (OLT) to evaluate the source memory, and the item memory was examined by a novel object recognition test (NORT). Some studies proved that what-where-when memory, namely episodic-like memory, is involved in cache recovery (Clayton and Dickinson, 1998). The scatter-hoarding could be considered as a what-where-when event that contains both source memory and item memory (Yi X et al., 2021). Combined with the enclosure data above, it meets our hypothesis that the mice which scatter hoarded more seeds have a better spatial memory. In this study, our results suggest that the SG possessed a superior long-term memory than the NG. The SG performed better in the OLT and NORT than the NG. This result proves our hypothesis that better memory is associated with the seed scatter-hoarding intensity of rodents. As rodents do not have eligible eyesight to relocate their cache positions, taking alternative strategies with the use of memory could be an evolutionary adaptation (Yi et al., 2016; Wang et al., 2018). In addition, the contrast performances in the two memory tasks were consistent with previous lesion studies that brain regions related to object location are segregated from regions associated with object recognition (Bussey et al., 1999; Mumby et al., 2002; Norman and Eacott, 2004; Barker et al., 2007). Yi X et al. (2021) have demonstrated that rodents pay more attention to the features of seeds to improve the effectiveness of future cache recovery of high-valued food items, which offers a possible explanation for the remarkable variation in NORT.

The role of gut microbiota in the regulation of brain function and behavior has been increasingly recognized (Leung and Thuret, 2015; Kennedy et al., 2016; Watanabe et al., 2021). The object recognition memory of the NG-SG group transplanted with microbiota from the SG group was significantly higher than that of the NG-NS group, providing the first evidence that gut microbiota may regulate the memory of scatter-hoarding rodents. It is consistent with the recent study that FMT from mice with Alzheimer’s disease inhibits neurogenesis by elevating colonic inflammation, then resulting in memory loss (Kim et al., 2021). We found FMT significantly increased the larder-hoarding intensity of the NG-SG group which is unexpected. FMT may stimulate the hoarding intensity of mice of both scatter hoarding (SH) and larder hoarding (LH). After FMT, the number of scatter-hoarded (SH) seeds in the NG-SG group was slightly higher than that in the NG-NS group, indicating FMT could increase the scatter-hoarding intensity. The non-significant FMT effect on scatter-hoarded seeds was likely caused by the relatively small sample size in enclosure conditions. Thus, these results generally support our hypothesis that FMT from donor mice with a high seed scatter-hoarding ability improved memory ability, except that the larder-hoarding intensity of the recipient mice was significantly increased in enclosure conditions. More work using large samples are needed to validate the FMT results in enclosures.

In addition, we found alpha and beta diversity analyses revealed that the gut microbiota structure of the NG-SG group is distinctively separated from the NG-NS group. Previous studies have confirmed that *Mucispirillum* spp. is common in rodents, but in small numbers and associated with a variety of diseases (Herp et al., 2021). *M. schaedleri* was found casually involved in Crohn’s disease-like colitis (Caruso et al., 2019). Meanwhile, a study using a mouse model of Crohn’s disease found that colitis can lead to depressive- and anxiety-like behaviors (Haj-Mirzaian et al., 2017). If this is true in our study, there is a possibility that FMT could induce colitis in recipient mice (NG-SG) and subsequently increase their anxiety and depression. It’s also possible that the anxious mice may adopt a more cautious approach in defending their caches, such as larder hoarding, which may explain the increased intensity of larder hoarding in NG-SG. However, *M. schaedleri* can also play a beneficial role in protecting the hosts from *Salmonella enterica* serovar Typhimurium-induced colitis (Herp et al., 2019). In this study, data demonstrated that *M. schaedleri* was enriched in the NG-SG group after FMT from the SG group. The increased abundance of *M. schaedleri* in the NG-SG group may be related to immunoreaction caused by FMT. In addition, *Lactobacillus* species are well-known probiotics for their anti-inflammatory and anti-oxidant effects, which can improve mood, synaptic ability, depression, and cognition (Desbonnet et al., 2008; Davari et al., 2013; Ruan et al., 2015). It was found that daily received *lactobacilli* (*L. rhamnosus, L. reuteri, and L. plantarum*) can prevent Lipopolysaccharide-induced (LPS-induced) elevated *TNF-α* mRNA expression in hippocampus and memory impairment (Zolfaghari et al., 2021). *TNF-α* is a proinflammatory cytokine that is associated with impaired brain function and numerous brain disorders (Masetto Antunes et al., 2022). It seems that the anti-inflammatory effect of *L. reuteri* prevents the memory deterioration. Moreover, it was recorded that the inoculation of *L. reuteri* F275 can promote the levels of the neurotransmitter, gamma-aminobutyric acid (GABA), in the hippocampus of the mice (Mao et al., 2020). Thus, the higher memory ability of the NG-SG group could be due to the elevated abundance of *L. reuteri*, correlating with increased levels of *TNF-α* and GABA.

In summary, our study revealed that scatter-hoarding behavior was closely related to spatial memory, and gut microbiota can modulate spatial memory and seed-hoarding behavior of rodents. It supports our hypothesis that gut microbiota is associated with spatial memory and seed-hoarding behavior of rodents via the gut-brain axis. Our results provide new insights into the potential role of gut microbiota in the study field of seed-hoarding behavior and memory of animals.

## Data availability statement

The datasets presented in this study can be found in online repositories. The names of the repository/repositories and accession number(s) can be found at: https://www.ncbi.nlm.nih.gov/, PRJNA974389.

## Ethics statement

The animal study was in accordance with the guidelines of Animal Use and Care Committee, Institute of Zoology, Chinese Academy of Sciences.

## Author contributions

ZZ and EF designed the experiments. EF, XY, KZ, and YL collected the data. EF, XY, and HZ analyzed the data. EF and XY wrote the first draft of the manuscript. ZZ and ZW revised the article. All authors contributed to the article and approved the submitted version.

## Funding

This study was supported by the National Natural Science Foundation of China (32001123, 32070460, and 32090021), and the Strategic Priority Research Program of the Chinese Academy of Sciences (XDB11050300).

## Conflict of interest

The authors declare that the research was conducted in the absence of any commercial or financial relationships that could be construed as a potential conflict of interest.

## Publisher’s note

All claims expressed in this article are solely those of the authors and do not necessarily represent those of their affiliated organizations, or those of the publisher, the editors and the reviewers. Any product that may be evaluated in this article, or claim that may be made by its manufacturer, is not guaranteed or endorsed by the publisher.
