# Supplementary material for: Gut microbiota is associated with spatial memory and seed-hoarding behavior of South China field mice (Apodemus draco)
Source: Front Microbiol. 2023 Sep 13;14:1236359. doi: 10.3389/fmicb.2023.1236359 (PMC10525317; doi:10.3389/fmicb.2023.1236359)
Supplement: Supplementary file 1 [file Data_Sheet_1.pdf]

Table S1. Numbers of mice used in each experiment and category

Period	Experiment	Category	Males	females	Total
Before FMT	Scatter-hoarding intensity test	SG	6	4	10
		NG	42	23	65
	Object location test[a]	SG	3	4	7
		NG	32	16	48
	Novel object recognition test[a]	SG	1	4	5
		NG	28	16	44
After FMT	Scatter-hoarding intensity test	NG-SG	11	10	21
		NG-NS	11	11	22
	Object location test[a]	NG-SG	8	9	17
		NG-NS	11	9	20
	Novel object recognition test[a]	NG-SG	9	9	18
		NG-NS	9	8	17
	16S rRNA	NG-SG	11	10	21
		NG-NS	11	11	22

[a]The mice which did not investigate the objects were excluded from the analyses

Abbreviations: FMT - fecal microbiota transplantation; SG - scatter-hoarding group; NG - non-scatter-hoarding group; NG-SG - non-scatter hoarding group administered with bacterial suspension from scatter-hoarding group; NG-NS - non-scatter hoarding group administered with sterile 0.9% saline.

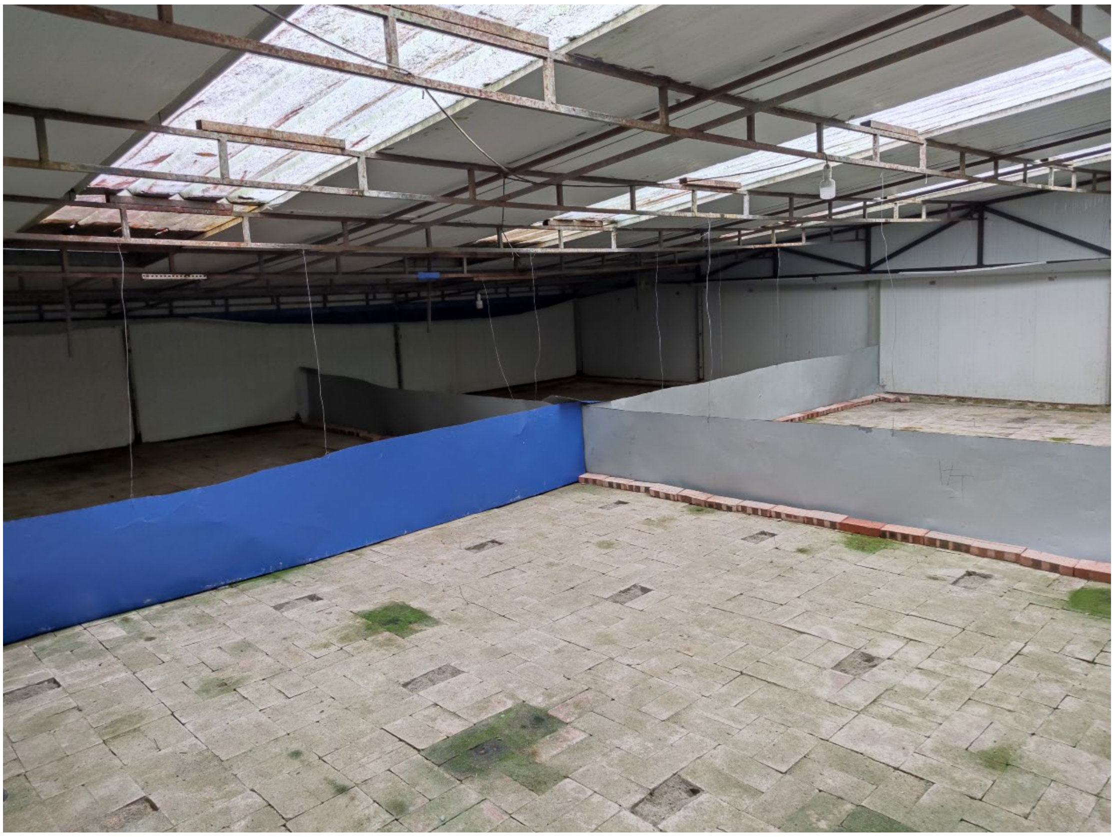

Fig. S1 Illustration of enclosures used in this study in the Dujiangyan City, Sichuan Province, China.

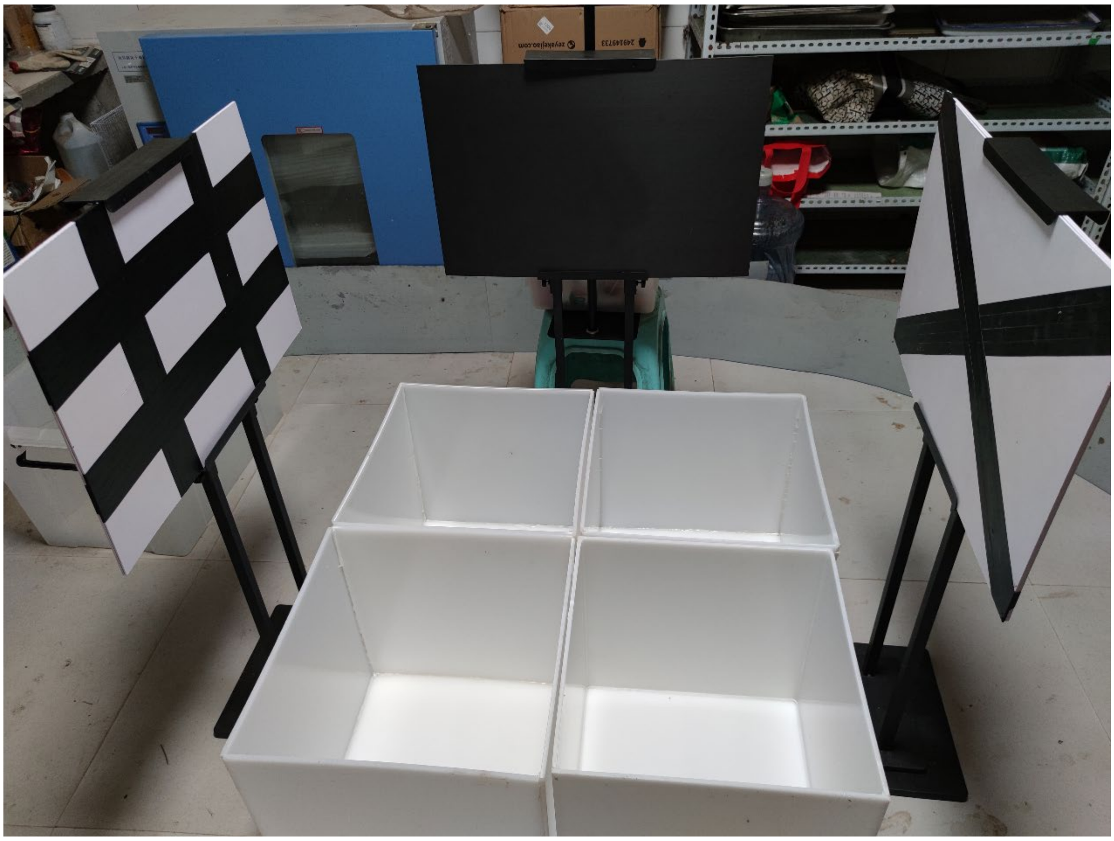

Fig. S2 Memory test arena assembly.

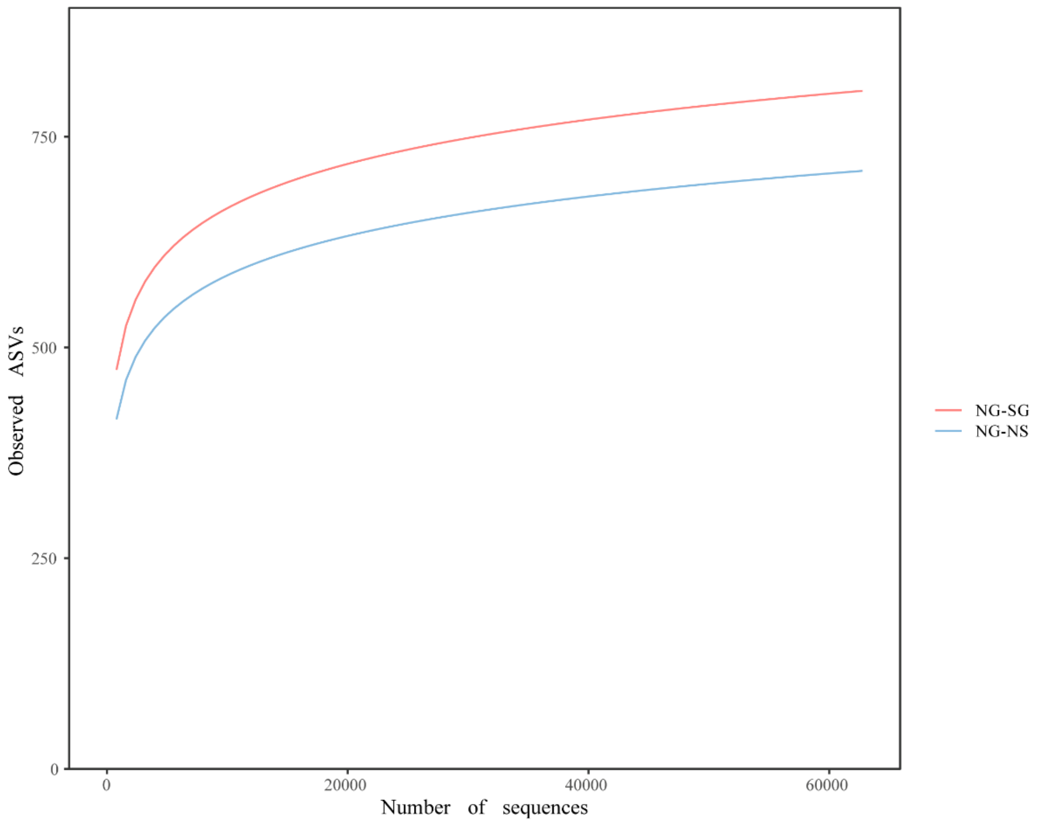

Fig. S3 Rarefaction curve of species diversity in mice of the non-scatter-hoarding group administered with either bacterial suspension from the scatter-hoarding group or sterile 0.9% saline (NG-SG and NG-NS, respectively). Abbreviation: ASVs - amplicon sequence variants.

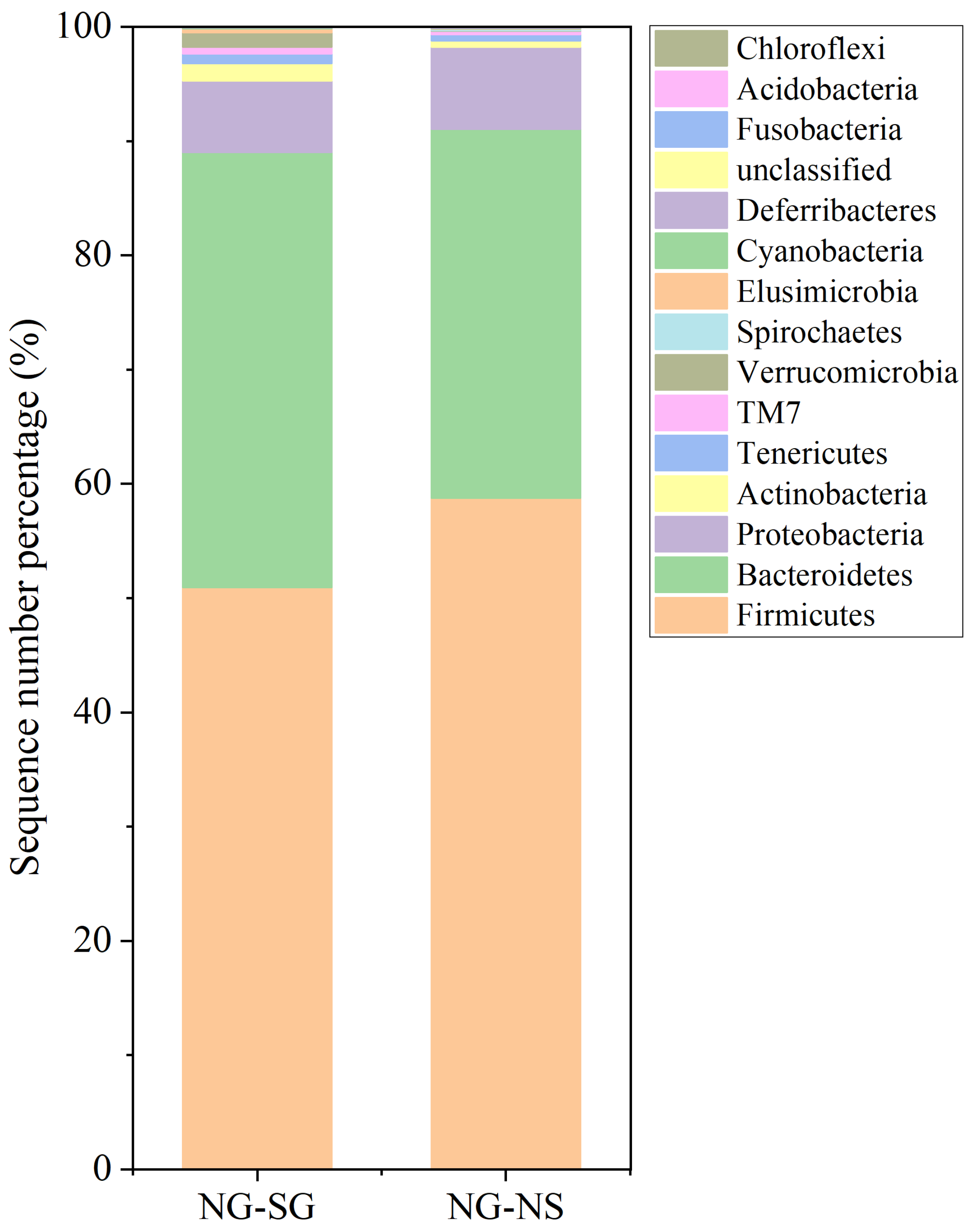

Fig. S4 Variation of gut microbial composition in mice of the non-scatter-hoarding group administered with either bacterial suspension from the scatter-hoarding group or sterile 0.9% saline (NG-SG and NG-NS, respectively).
